# Wing tucks are a response to atmospheric turbulence in the soaring flight of the steppe eagle *Aquila nipalensis*

**DOI:** 10.1098/rsif.2014.0645

**Published:** 2014-12-06

**Authors:** Kate V. Reynolds, Adrian L. R. Thomas, Graham K. Taylor

**Affiliations:** Department of Zoology, University of Oxford, Oxford OX1 3PS, UK

**Keywords:** soaring, wing tuck, bird flight, gust response, gust alleviation, atmospheric turbulence

## Abstract

Turbulent atmospheric conditions represent a challenge to stable flight in soaring birds, which are often seen to drop their wings in a transient motion that we call a tuck. Here, we investigate the mechanics, occurrence and causation of wing tucking in a captive steppe eagle *Aquila nipalensis*, using ground-based video and onboard inertial instrumentation. Statistical analysis of 2594 tucks, identified automatically from 45 flights, reveals that wing tucks occur more frequently under conditions of higher atmospheric turbulence. Furthermore, wing tucks are usually preceded by transient increases in airspeed, load factor and pitch rate, consistent with the bird encountering a headwind gust. The tuck itself immediately follows a rapid drop in angle of attack, caused by a downdraft or nose-down pitch motion, which produces a rapid drop in load factor. Positive aerodynamic loading acts to elevate the wings, and the resulting aerodynamic moment must therefore be balanced in soaring by an opposing musculoskeletal moment. Wing tucking presumably occurs when the reduction in the aerodynamic moment caused by a drop in load factor is not met by an equivalent reduction in the applied musculoskeletal moment. We conclude that wing tucks represent a gust response precipitated by a transient drop in aerodynamic loading.

## 1. Introduction

**Figure 1.**
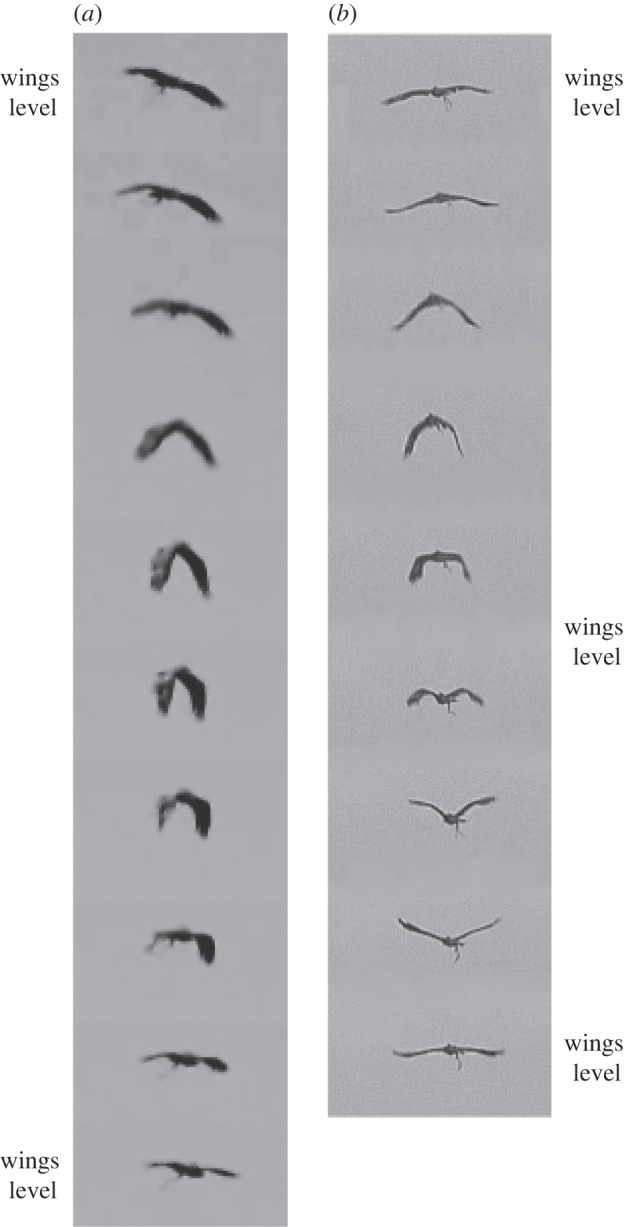
(*a*) Sequence of video frames showing a typical wing tuck. (*b*) Sequence of video frames showing a typical wingbeat, which is easily distinguishable from a tuck on account of its shorter period, and the fact that the wings are raised above the body before being returned to a level configuration. Time interval between frames: 0.033 s. Complete animations of these video sequences are provided in the electronic supplementary material, videos S1 and S2.

One of the most impressive features of birds is their ability to fly in atmospheric conditions that would keep a light aircraft grounded. Most recent research on avian flight stability has treated the airframe as fixed [1–7], but birds differ fundamentally from fixed-wing aircraft in the way in which their flexible, neuromuscularly controlled airframe responds to atmospheric turbulence. As Wright [8] first observed, a bird pitched up by a gust ‘immediately lowers its wings much below its body’. We call this type of motion a wing tuck [9], which we define as a manoeuvre in which a soaring bird rapidly pulls its wings down beneath the level of the body, before spreading them back up into a gliding configuration. This motion (see the electronic supplementary material, video S1) is distinct from a wingbeat (see the electronic supplementary material, video S2), because the wings are not raised above the level of the body during a wing tuck (figure 1*a,b*). Furthermore, the extent to which the wings are pulled down beneath the body varies considerably between tucks. Wing tucks are a common and phylogenetically widespread behaviour among large soaring birds, having been described in New World vultures (Cathartidae) [8], Old World vultures, kites and eagles (Accipitridae) [9,10] and storks (Ciconiidae) [10]. Indeed, wing tucks are a sufficiently common behaviour in certain species of soaring bird, especially the New World and Old World vultures, that they are mentioned as a taxon-specific identification character in some ornithological field guides [11–13].

Birds fly predominantly in the surface boundary layer of the atmosphere, where turbulence is strongly influenced by surface conditions, including terrain and temperature [14]. There are two main sources of atmospheric turbulence: mechanical and thermal. Mechanical turbulence is caused by friction as air flows over the surface of the Earth. Wind speeds decrease nearer the surface as the effects of surface friction increase, which creates wind shear [14]. This shear produces rotating air motions or eddies on a large scale: other things being equal, the greater the wind speed, the greater the mechanical turbulence that is generated. Other factors influencing mechanical turbulence include the surface roughness, and the height of obstructions which deflect and mix the airflow [15]. By contrast, thermal turbulence is caused by buoyancy effects [15]. Under unstable atmospheric conditions, uneven heating of the Earth's surface generates large circulation systems called thermals, where rising warm air is replaced by colder air drawn in at the bottom of the thermal column. Thermal turbulence increases with the intensity of surface heating and the degree of atmospheric instability, which is related to temperature lapse rate [16]. In addition, thermals actually magnify the vertical mixing caused by mechanical turbulence [15]. These two distinct sources of atmospheric turbulence present obvious challenges to flight stability and control, but they also provide the opportunity and energy source for soaring flight. It follows that soaring birds have to be able to cope with turbulence in order to soar, and we hypothesize that their wing tucks represent a response to this.

Wing tucks have previously been interpreted as a deliberate mechanism for restoring pitch equilibrium following a gust perturbation [8], as an active mechanism for gaining airspeed prior to a flat glide or dive [10], and as an active mechanism for alleviating gust loads [9,17]. However, none of these hypotheses has yet been tested conclusively. Here, we use ground-based video and onboard inertial instrumentation [9] to analyse the mechanics, occurrence and causation of wing tucking in the steppe eagle *Aquila nipalensis* (Hodgson)—a typical thermal-soaring migrant [18–20]. We use these data to detect the occurrence of wing tucks automatically, and to identify the changes in the motion state of the bird that characteristically precede and accompany wing tucks. We then use standard model selection techniques [21] to investigate the statistical relationship between the rate of wing tucking and several environmental predictors of atmospheric turbulence. This analysis allows us to identify the environmental parameters that best predict the rate at which wing tucking occurs in flight. Finally, we analyse the causation of wing tucking, using our inertial measurements to do so. Together, these analyses provide strong evidence that wing tucks occur in response to atmospheric turbulence, as well as offering insight into the mechanistic and functional basis of wing tucking.

## 2. Material and methods

### 2.1. Experiments

**Figure 2.**
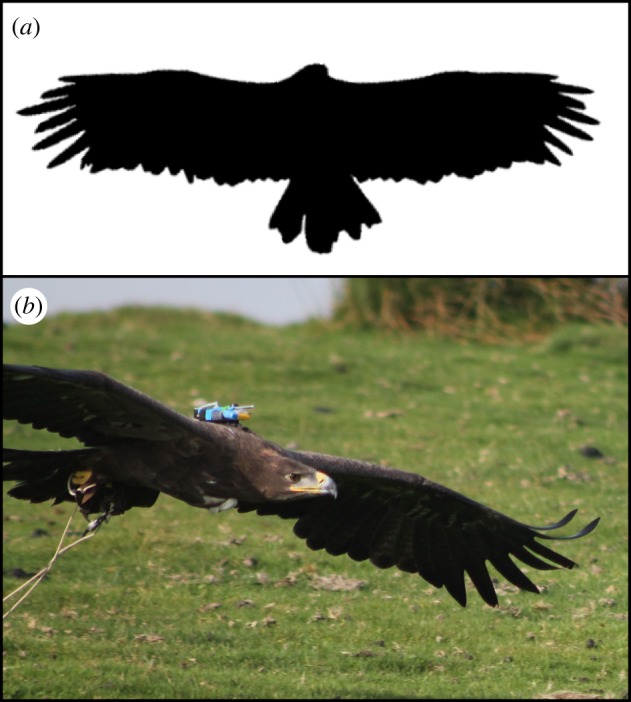
(*a*) Thresholded digital photograph of the bird's planform taken from directly below when the bird was gliding in a typical soaring posture with its wings fully outstretched (wing span = 1.9 m, from tip to tip). The irregular outline of the wings and tail reflects the state of the feathers, some of which were recently moulted. (*b*) Photograph of bird showing instrumentation unit mounted dorsally.

We used the same trained, captive, male steppe eagle over 45 separate flight tests (see table 1 for morphological measurements). The bird's body mass was measured daily during the flight season; other morphological measurements were made using ImageJ (National Institutes of Health, Bethesda, MD) from a digital photograph of the bird's planform in flight with the wings outstretched in a typical soaring configuration (figure 2*a*). The image was scaled against measurements of arm wing chord made in flight in a previous study using multi-station photogrammetry [22].

**Table 1.** Morphological measurements of the steppe eagle used in this study. Body mass was measured daily, and its range reflects the range of variation over the course of the study. Wing area, wing span and aspect ratio are given for a typical soaring flight configuration with the wings fully outstretched (figure 2).

morphological parameter	measurement
body mass	2.25–2.40 kg
aspect ratio	6.7
wing area	0.54 m^2^
wing span	1.9 m

The bird was flown at several sites in the Black Mountains region of South Wales, UK, during the summer of 2012. Sites were selected according to wind direction to provide a soarable ridge line facing into the wind on the day of the test. The bird was flown under a wide range of atmospheric conditions, but was not flown in rain, or in winds exceeding 8 ms^−1^. The flights, which lasted between 10 and 50 min, all involved ridge soaring or thermalling behaviour. The bird was filmed with a Sony HDR-SR1 high-definition digital video camera. A WindMaster Pro three-axis ultrasonic anemometer (Gill Instruments Ltd, Lymington, UK) was used to obtain measurements of local wind velocity. The anemometer was positioned at the top of the ridge, approximately 2 m above the ground, and measured three-axis velocities at 10 Hz.

An onboard instrumentation package was mounted dorsally on the bird prior to each flight using a removable harness with Teflon ribbon straps (Marshall Direct Ltd, Pennington Leigh, UK). The harness was worn in a rucksack-like arrangement over the back and chest, with the instrumentation positioned as close to the centre of gravity as possible (figure 2*b*). The experimental protocol was approved by the United States Air Force, Surgeon General's Human and Animal Research Panel and by the University of Oxford, Department of Zoology, Local Ethical Review Committee, and was considered not to pose any significant risk of causing pain, suffering, damage or lasting harm to the animal involved.

### 2.2. Instrumentation

The onboard instrumentation comprised an ArduPilotMega2 (3D Robotics, San Diego, CA) board running customized software, containing an inertial measurement unit (IMU), global positioning system (GPS), thermometer, barometer and pitot tube. The equipment helped up to 50 min of continuous recording time and weighed less than 0.075 kg with battery. This represents less than half the range of natural variation in body mass, and was always less than 3.3% of body mass (table 1). Observations of flights with and without onboard instrumentation indicate that the bird adapted easily to the additional loading, which made no notable difference to flight performance—even in turbulent conditions. The IMU-logged measurements of three-axis linear acceleration, angular rotation rate and Earth magnetic field data at 50 Hz, defined in a right-handed axis system fixed with respect to the measurement unit, with the *x*-axis aligned approximately with the longitudinal axis of the bird's body pointing forwards, and the *z*-axis pointing ventrally. Position, altitude and ground speed were obtained at 10 Hz from the GPS. Temperature, barometric pressure and airspeed were also recorded at 10 Hz. Airspeed was calculated from the differential pressure measured by the pitot tube, using a calibration factor that was determined in a wind tunnel prior to field tests. The mean airspeed of 14.5 ms^−1^ that we recorded is reassuringly close to the mean airspeed of 15.6 ± 0.32 ms^−1^ (mean ± s.e.) measured in a previous radar tracking study of *n* = 52 migrating steppe eagles [18].

The performance specifications of the sensors which we use in this study are given in table 2. During a typical wing tuck, the wings are returned to their initial position within approximately 0.35 s of the time at which they begin to drop (figure 1*a*; electronic supplementary material, video S1). This is 3.5 times the sampling interval of the airspeed measurements, and 17.5 times the sampling interval of the inertial measurements, which demonstrates that the sampling rate of the instrumentation is more than sufficient to characterize even this quickest portion of the tuck manoeuvre. The transient body motions that are associated with wing tucking occur over a much longer period (more than 6 s), so will obviously be well characterized at this sampling rate.

**Table 2.** Specifications of the sensors used in this study. Noise characteristics are given according to the manufacturers' specifications, except for the pitot tube, which we characterized ourselves in a low-turbulence wind tunnel.

sensor	sampling rate (Hz)	units	resolution	noise	noise characteristic
accelerometers	50	ms*^−^*^2^	2.4 × 10*^−^*^3^	3.9 × 10*^−^*^3^ 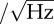	spectral density (at 10 Hz)
rate gyros	50	°s*^−^*^1^	6.1 × 10*^−^*^2^	5.0 × 10*^−^*^3^ 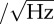	spectral density (at 10 Hz)
pitot tube	10	kPa	5.8 × 10*^−^*^3^	<7.9% RMS	full bandwidth (at 0.03 kPa)
anemometer	10	ms*^−^*^1^	1.0 × 10*^−^*^3^	<1.5% RMS	full bandwidth (at 12 ms^−1^)

### 2.3. Data processing

Although the inertial instrumentation was positioned as close as possible to the average position of the centre of mass of the bird, it could not have coincided exactly, because the centre of mass falls inside the bird's body. It follows that the accelerations sensed by the accelerometers during rigid body motion would have included additional contributions owing to the angular velocity and angular acceleration of the body, proportional to the displacement of the accelerometers from the centre of mass. The accelerations {*a_x_*, *a_y_*, *a_z_*} sensed at an arbitrary point on a rigid body are2.1
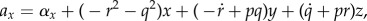
2.2
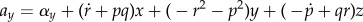
2.3
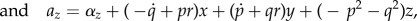
where {*α_x_*, *α_y_*, *α_z_*} is the acceleration at the centre of mass, {*p*, *q*, *r*} is the angular velocity, 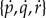 is the angular acceleration and {*x*, *y*, *z*} is the displacement of the IMU from the centre of mass [23]. These equations are linear in {*x*, *y*, *z*}, and the IMU's average displacement from the bird's centre of mass was therefore estimated from a least-squares solution of these equations using the inertial measurements themselves (see the electronic supplementary material, methods and [9]). The resulting parameter estimates were then used to correct the measurements of the sensed acceleration, so as to reduce the effect of any systematic measurement errors introduced by the placement of the IMU.

The instantaneous total load factor (*N*_t_, colloquially the ‘g-load’ experienced by the bird) was defined as the total acceleration at the centre of mass divided by the gravitational acceleration (*g* = 9.81 ms^−2^)2.4
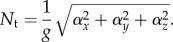
The load factor is a standard quantity in aeronautics and has the useful property that *N*_t_ = 1 at equilibrium, when the total aerodynamic force vector is equal and opposite to the weight of the bird (i.e. directed vertically).

At shallow glide angles, the lift vector (i.e. the component of the total aerodynamic force vector that is perpendicular to the relative airflow) is itself close to the vertical. In this case, the load factor is approximately equal to the instantaneous lift of the bird (*L*) divided by its body weight (*W*). We may therefore use the approximation *L* ≈ *N*_t_*W* to rewrite the standard lift equation2.5
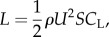
as2.6
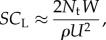
where *ρ* is the air density, *U* is the airspeed, *S* is the total lifting surface area and *C*_L_ is the mean section lift coefficient. The quantity *SC*_L_ is a function of flight morphology and angle of attack, and so measures the net effect of the bird's instantaneous flight configuration on the lift that it produces. Hence, although we are unable to use our measurements of *SC*_L_ to separate the effects of changing wing and tail area from the effects of changing wing and tail angle of attack, we are able to use our measurements of *SC*_L_ to identify the net effect of these control inputs upon the bird's flight. It should be noted, however, that the approximation in equation (2.6) breaks down if the bird is not in a shallow glide.

### 2.4. Automatic tuck identification

**Figure 3.**
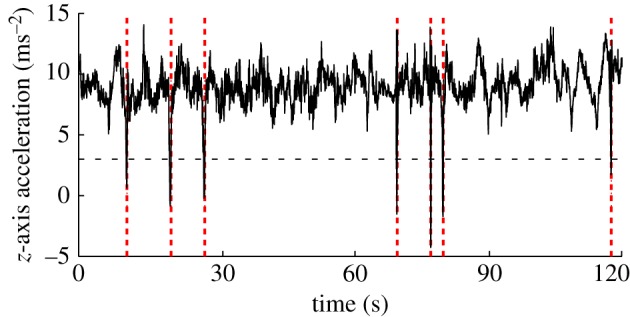
Plot of measured *z*-acceleration against time for one randomly chosen section of flight lasting 2 min. The seven wing tucks (vertical dashed lines) occurring in this sequence were identified automatically as sections of flight for which the correlation with the tuck template (figure 4) was >0.45, and during which the magnitude of the *z*-acceleration dropped below 3 ms^−2^ (horizontal dashed line). (Online version in colour.)

**Figure 4.**
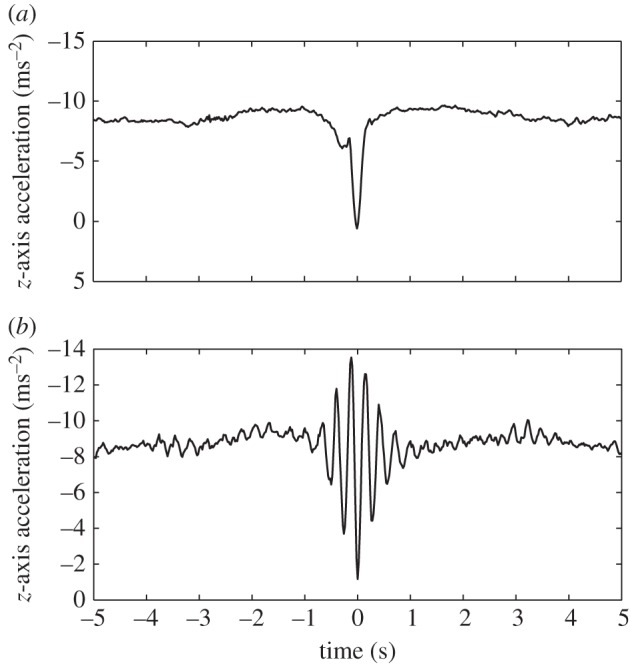
(*a*) Tuck template, defined as the time history of the *z*-acceleration (*α_z_*), averaged over the 80 tucks which we manually identified from the ground-based video, aligned with respect to the peak in *α_z_* at *t* = 0 s. (*b*) Flapping template, defined as the time history of *α_z_*, averaged over 69 manually identified wingbeats and aligned similarly. Because wingbeats rarely occur singly, the template shows multiple peaks, each representing a single wing beat; the attenuation of the wingbeats to either side of the middle wingbeat is an effect of the averaging method used to construct the template, but has no adverse effect upon wingbeat identification. Wingbeat frequency is approximately 3 Hz. Note that, in contrast to the tuck template, the wingbeat template shows approximately symmetric, rather than one-sided, changes in *α_z_*.

Wing tucks were identified automatically in Matlab from the inertial data that we collected, using a method first developed in [17] but explained fully below. We used the *z*-acceleration traces (figure 3) as the basis of this identification procedure, because the dorsoventral acceleration of the bird provides a reliable indicator of whether the wings are open or closed. In the first instance, we manually identified 80 tucks from approximately 20 min of video data, sampled over 6 days. The inertial measurements corresponding to these manually identified tucks were then used to construct a template for the accompanying pattern of changes in the *z*-acceleration sensed by the IMU (figure 4*a*). We aligned the *z*-acceleration traces for each manually identified tuck with respect to the point of peak *z*-acceleration, taking this reference to define the time *t* = 0 s. The mean instantaneous *z*-acceleration over all of the aligned tucks was then used to form the template.

Wing tucks were identified automatically by searching the data for peaks in the cross-correlation function between the tuck template and the *z*-acceleration data sensed by the IMU. Peaks in the cross-correlation function occurring within 0.25 s of each other were assumed to represent a single tuck, with the chosen interval corresponding to the width in the template of the spike in the *z*-acceleration (figure 4). We identified tucks as sections of flight for which the correlation with the tuck template was more than 0.45, and during which the magnitude of the *z*-acceleration dropped below 3 ms^−2^. In arriving at this combination of threshold values, we tested the automatic tuck identification algorithm on the sections of data that we had analysed manually, with the objective of maximizing the number of wing tucks that the algorithm identified while keeping the number of false positives zero. Having optimized the algorithm accordingly, our automatic procedure successfully identified more than 80% of the tucks that we had identified ourselves from the 20 min of video data that we analysed manually. Thus, we chose threshold values that gave a sample type I error rate of 0.8 and a sample type II error rate of zero when automatically detecting the wing tucks that we had identified manually during these 20 min of video data.

To ensure that occasional wingbeats were not mistaken for wing tucks, a flapping template (figure 4*b*) was created using the same method, and the results were compared. Any ambiguous records were checked manually by exploring the raw inertial data, and were easily distinguished as either a wingbeat or a wing tuck (compare figure 4*a* and *b*). In total, we identified 2594 tucks over the entire dataset, giving an overall identified tuck rate of approximately 2.2 identified tucks per minute. Given that the thresholds that we set for the automatic tuck-identification algorithm resulted in a sample type I error rate of 0.8 for the manually analysed video data, the true tuck rate is expected to have been closer to 2.7 tucks per minute. In any case, it is clear that wing tucks are a common feature of soaring flight in our steppe eagle.

### 2.5. Environmental predictors of atmospheric turbulence

In order to test whether the rate of wing tucking was related to atmospheric turbulence, we computed the identified tuck rate for each flight (*f*) and defined several environmental predictors of atmospheric turbulence that could be measured for each flight. Other things being equal, we would expect the level of mechanical turbulence to increase with increasing mean wind speed, as a result of the greater wind shear strength (see Introduction). Mean wind speed estimates (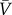) were obtained from the Met Office Integrated Data Archive System (MIDAS) land and marine surface station data [24] for the Credenhill weather station near Hereford, UK. At an altitude of 76 m, this weather station is 21.6 km from the nearest flying site and 54.0 km from the furthest, so the MIDAS measurements only provide a general indication of how windy it was during a given flight. We averaged the three hourly MIDAS recordings closest to the time at which the bird flew, to give an estimate of the mean wind speed for each flight. We also included our anemometer measurements of the mean local wind speed on the ridge (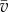) in the analysis. The fluctuating local wind velocity components on the ridge provide a measure of the atmospheric turbulence levels in the general locality of the flight, which were summarized by the standard deviation of the anemometer readings (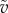). The MIDAS measurements of mean wind speed and the anemometer measurements of local wind speed on the ridge therefore characterize the wider atmospheric conditions pertinent to a given flight, and should not be interpreted as estimates of the mean wind speed or turbulence at the precise time and location at which the bird was flying.

The mean altitude above sea level (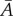) at which the bird flew was computed from the onboard GPS altitude log. Flight altitude is dependent on the height of the terrain, but other things being equal, the bird is expected to have flown higher on days with greater thermal activity, and hence greater buoyant production of turbulence. Furthermore, the structure of mechanical turbulence changes with altitude, as eddy size becomes larger with increasing altitude, and so, for both reasons, we would expect mean altitude above sea level to be positively correlated with mean atmospheric turbulence levels. The size of the orographic obstruction generating this mechanical turbulence was approximated as the height above sea level (*r*) of the ridge where the bird was flown, which was taken from Ordnance Survey maps; the larger the obstruction, the greater the wind shear, and hence the higher the expected level of mechanical turbulence. Regional Atmospheric Soaring Prediction (RASP), a forecasting tool designed for glider pilots to evaluate soaring conditions [25], was also used to estimate thermal updraft strength, averaged over the three predictions closest to the time of flight.

## 3. Results

### 3.1. Mechanics of wing tucking

**Figure 5.**
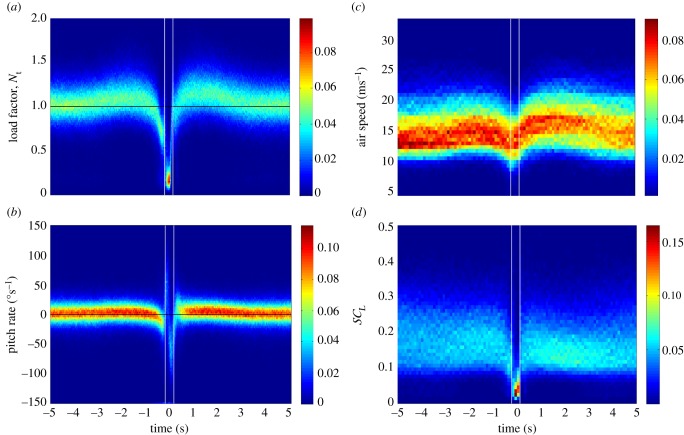
Two-dimensional histograms for all 2594 automatically identified tucks showing the temporal variation in the frequency density of (*a*) instantaneous total load factor, *N*_t_; (*b*) pitch rate *q*, signed positive in a nose-up direction; (*c*) airspeed, *U*; and (*d*) *SC*_L_, representing the product of wing area (*S*) and lift coefficient (*C*_L_). All times are referenced to the point of minimum load factor *N_z_* = *−α_z_*/*g* for each tuck at *t* = 0 s, as calculated from the *z*-component of acceleration (*α_z_*). The colour bars to the right of each histogram relate the colour of each point on the histogram to the proportion of the sampled tucks at that time to which it corresponds. The data were binned into 100 bins for load factor and pitch rate, which were sampled at 50 Hz; 50 bins were used for airspeed and *SC*_L_, which were sampled at a lower rate of 10 Hz. The approximate beginning and end of the tucking movement of the wings, inferred from the discontinuities in the distributions for load factor and *SC*_L_, are denoted by the vertical white lines.

**Figure 6.**
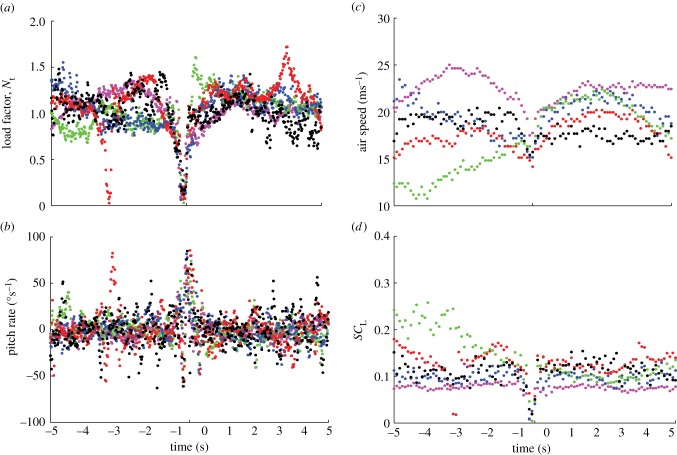
Data for five individual tucks randomly selected from the 2594 automatically identified tucks, showing (*a*) instantaneous total load factor, *N*_t_; (*b*) pitch rate *q*, signed positive in a nose-up direction; (*c*) airspeed, *U*; and (*d*) *SC*_L_, representing the product of wing area (*S*) and lift coefficient (*C*_L_).

The video sequences in figure 1 show a typical wing tuck (figure 1*a* and the electronic supplementary material, video S1), and a typical wingbeat for comparison (figure 1*b* and the electronic supplementary material, video S2). The bird starts the tuck with its wings outstretched in the gliding position. It then begins to drop its wings down beneath the body by rotating them leading-edge down about the shoulder joint. The wings are held in their dropped position momentarily before the recovery stroke, in which the wings are rotated leading-edge up, brought in and raised back to the level of the body. Thus, whereas the wings are raised above the level of the body during a wingbeat, this does not happen during a wing tuck. Nevertheless, the time taken for the whole tuck is about 50% longer than the time taken to go from wings level to wings level during the equivalent half-stroke in a normal wingbeat (figure 1). The accompanying changes in total load factor, airspeed, pitch rate and *SC*_L_ are summarized for all 2594 identified tucks in the two-dimensional histogram plots shown in figure 5, which represent the frequency density of the values taken by each variable at each time step over a period of 5 s either side of time *t* = 0 s (i.e. over a 10 s window beginning 5 s before the point of peak *z*-acceleration). Figure 6 plots the time-series data from five randomly selected tucks to provide an indication of how the pattern of variation during a single wing tuck reflects the overall pattern of variation shown in the two-dimensional histograms (figure 5).

In the period leading up to a wing tuck, the bird typically averages close to an equilibrium flight condition, with a mean instantaneous load factor of *N*_t_ ≈ 1 (figure 5*a*) and a mean instantaneous pitch rate of approximately zero at *t* = −5.0 s (figure 5*b*). The wing tuck itself typically occurs between *t* = −0.225 and *t* = 0.125 s, as is obvious from the discontinuity and drop-off in the *SC*_L_ plot (figure 5*d*), and also from the corresponding decline in total load factor at this time (figure 5*a*). These changes in wing loading are accompanied by a distinct drop in airspeed (figure 5*c*) and by a sharp nose-down pitching motion (figure 5*b*). The wings are reopened shortly after *t* = 0 s, whereupon loading is rapidly re-established (figure 5*a*), airspeed increases (figure 5*c*) and the bird starts to pitch nose-up again (figure 5*b*). The 0.35 s duration of the tuck estimated from the inertial data (figure 5) closely matches the duration of the wing tucking movement seen in the video sequence in figure 1*a*. Following the tuck, there is typically a second surge in airspeed (figure 5*c*) and load factor (figure 5*a*), which occurs between *t* ≈ 0.125 and *t* ≈ 3 s. By *t* ≈ 5 s, the bird has returned to an equilibrium flight condition with *N*_t_ ≈ 1 (figure 5*a*) and a mean instantaneous pitch rate of approximately zero (figure 5*b*). On average, *SC*_L_ tends to be slightly lower after the tuck than before (figure 5*d*), which implies that angle of attack and/or wing area are decreased slightly following a tuck. This is associated with the slight gain in airspeed that is apparent in figure 5*c*.

**Figure 7.**
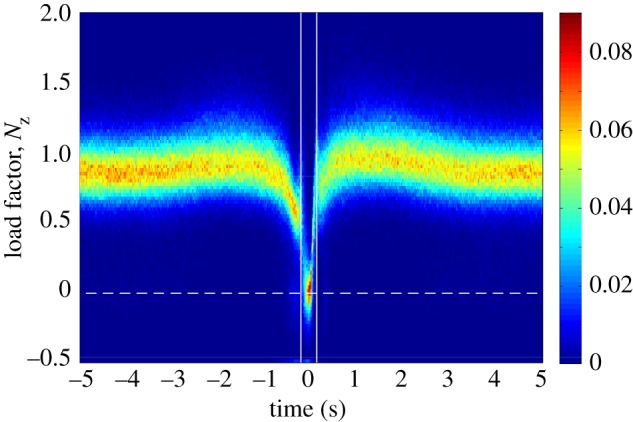
Two-dimensional histogram for all 2594 automatically identified tucks, with 100 bins showing the temporal variation in the frequency density of the instantaneous load factor *N_z_* = *−α_z_*/*g* as calculated from the *z*-component of acceleration (*α_z_*). All times are referenced to the point of minimum load factor *N_z_* for each tuck at *t* = 0 s. The colour bar relates the colour of each point on the histogram to the proportion of the sampled tucks at that time to which it corresponds. The approximate beginning and end of the tucking movement are denoted by the vertical white lines, as in figure 5. The dashed horizontal white line denotes zero load factor.

The total load factor *N*_t_ did not usually drop to zero during a wing tuck (figure 5*a*). However, *N*_t_ is calculated from the magnitude of the total acceleration vector, independent of its direction, and is therefore constrained to be non-negative by definition (equation (2.4)). Hence, we cannot tell directly from figure 5*a* whether the wings actually experienced load reversal at any point during the tuck. We therefore examined the component of the total load factor calculated from the *z*-acceleration component, *N_z_* = −*α_z_*/*g* (figure 7). This quantity does not measure the total aerodynamic load experienced by the bird, and, indeed, its equilibrium value was less than 1 at *t* = −5 s (figure 7), which indicates that the *z*-axis of the IMU was not quite vertical at equilibrium. Nevertheless, *N_z_* is generally signed positive if the wings are generating a dorsally directed aerodynamic force, and signed negative if the wings are generating a ventrally directed aerodynamic force. The minimum value of *N*_z_ during a tuck was centred on zero, which suggests that the wings may sometimes have experienced a slight negative aerodynamic loading, and that the bird approached a momentary state of free fall when its wings were dropped.

In order to identify the probable cause of wing tucking, we looked at the IMU data corresponding to the 5 s period leading up to the point of peak *z*-acceleration at *t* = 0. Although the airspeed data are noisy, there was usually a distinct surge in airspeed between *t* = −5 and −1.5 s (figure 5*c*). This was accompanied by an increase in total load factor (figure 5*a*), and by a nose-up pitching motion of approximately 5° amplitude, estimated by integrating the mean measured pitch rate (figure 5*b*). Airspeed, total load factor and pitch rate all began to fall off at an increasing rate from *t* ≈ −1.5 s, and were already in sharp decline by *t* ≈ −0.7 s, roughly 0.45 s before the wings had started to tuck (figure 5). We therefore conclude that these changes in airspeed, total load factor and pitch angle cannot possibly have been caused by tucking of the wings. Instead, they may very well have been its immediate cause.

We may explore the relationship between total load factor and airspeed further by rearranging equation (2.6) to yield3.1
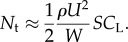
Because air density (*ρ*) and body weight (*W*) are effectively constants, and because *SC*_L_ does not increase before the wings begin to tuck (figure 5*d*), it is clear by inspection of equation (3.1) that the initial surge in load factor must have been caused by the accompanying surge in airspeed (*U*). Furthermore, because there is no indication of any consistent change in *SC*_L_ before the tuck, this surge in airspeed must have been caused by atmospheric perturbation, rather than by deliberate changes in airspeed on the part of the bird.

### 3.2. Environmental predictors of wing tucking

If wing tucking occurs in response to atmospheric perturbation, we would expect the rate of wing tucking to be positively related to the level of atmospheric turbulence. We used model selection techniques to investigate the statistical relationship between the square root transform [26] of the rate of wing tucking, 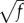, and several environmental predictors of atmospheric turbulence. A candidate set of 29 ordinary least-squares models were created from all possible linear combinations of MIDAS mean wind speed (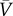), mean local wind speed on the ridge (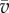), standard deviation of the local wind speed on the ridge (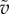), the interaction (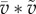) and either mean flight altitude (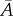) or ridge height (*r*). Average flight altitude (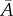) and ridge height (*r*) were highly correlated with each other (Pearson's correlation coefficient: 0.94), so we did not combine these predictors in any one model, to avoid the problems of multi-collinearity that would have resulted from combining them in a single model. RASP thermal predictors explained almost none of the variation in tuck rate (e.g. *R*^2^ = 0.072 for the regression of 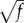 on the predicted thermal height), so were not considered further here.

The second-order Akaike information criterion (AIC_c_) was used to rank the relative performance of the competing candidate models. This is a standard approach to model selection with a rigorous information-theoretic interpretation [21], but has been largely overlooked in the biomechanics literature to date. AIC_c_ was calculated for each model as3.2
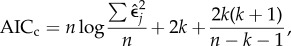
where 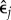 denotes the estimated residuals, *n* the sample size and *k* the number of estimated parameters. Under this criterion, the best model within the candidate set is the one which minimizes AIC_c_. The criterion therefore favours models with higher predictive power, but penalizes this according to the number of parameters that are estimated in fitting the model. The relative performance of the other models is expressed as the difference (Δ*_i_*) between their AIC_c_ value and that of the best model. This difference can be used to compute the likelihood of each model given the data [21], which is proportional to 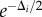. Thus, the best model has the highest likelihood given the data. The 15 most highly ranked candidate models are shown in table 3. The relative likelihood of each of the candidate models is summarized by their Akaike weights (*w_i_*) in table 3, calculated by normalizing the likelihoods of the models given the data, so that they sum to one [21].

**Table 3.** Top 15 candidate models predicting the square root transform of the identified tuck rate (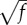), ranked by their AIC_c_ values, and rated by their ΔAIC_c_ values (Δ*_i_*) and Akaike weights (*w_i_*). The 95% confidence set, defined as the smallest set of models whose Akaike weights *w_i_* sum to ≥0.95, contains the top four models. 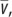 mean local wind speed on the ridge; 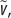 standard deviation of local wind speed on the ridge; 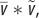 interaction between mean and standard deviation of local wind speed on the ridge; 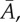 mean flight altitude; *r*, ridge height; 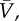 mean MIDAS wind speed.

model parameters	AIC_c_	Δ*_i_*	*w_i_*
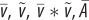	−90.556	0.000	0.612
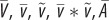	−88.242	2.314	0.192
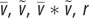	−87.285	3.271	0.119
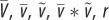	−84.460	6.096	0.029
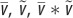	−83.993	6.563	0.023
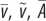	−81.914	8.642	0.008
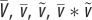	−81.315	9.241	0.006
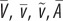	−79.901	10.655	0.003
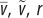	−79.648	10.908	0.003
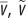	−78.598	11.958	0.002
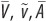	−77.446	13.110	0.001
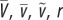	−77.155	13.401	0.001
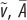	−77.062	13.494	0.001
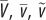	−76.213	14.343	0.000
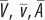	−74.484	16.072	0.000

The best approximating model for these data (top row of table 3) includes the mean local wind speed on the ridge (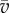), the standard deviation of the local wind speed on the ridge (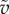), their interaction (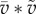) and the mean flight altitude (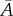). The estimated regression coefficients for this model, and their associated conditional standard errors are shown in table 4 (*F*_5,34_ = 19.59; 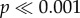; *R*^2^ = 0.697). Several of the other models also perform well, however, and the 95% confidence set, defined as the smallest set of models whose Akaike weights sum to 0.95, includes three other models. The evidence ratios, calculated as the ratios of their Akaike weights to that of the best model, are 3.2, 5.1 and 21.1, respectively, so it is quite likely that the second and third models might become top-ranked if replicate samples of data were available [21]. In any case, our anemometer measurements of the mean 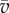 and standard deviation 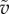 of the local wind speed on the ridge, and the interaction between them 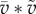, appear in all of the models within the 95% confidence set. We therefore infer that local atmospheric conditions are the most important predictors of tuck frequency, which is precisely as expected if wing tucking occurs in response to atmospheric perturbation.

**Table 4.** Parameter estimates for the best candidate model predicting the square root transform of the identified tuck rate (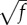), giving the regression coefficient estimates and associated conditional standard errors for each term.

parameter	intercept	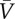	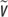	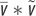	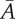
regression coefficient	−1.78	0.290	2.16	−0.223	1.04 × 10^−3^
standard error	0.513	6.80 × 10^−2^	0.485	6.56 × 10^−2^	3.42 × 10^−4^

## 4. Discussion

### 4.1. Wing tucks as a form of gust response

Our two analyses—of the mechanics of wing tucking, and of the environmental predictors of tuck rate—each provide independent support for the hypothesis that wing tucks occur in response to atmospheric turbulence. The former analysis demonstrates that wing tucks occur following a surge in load factor explained by a transient increase in airspeed caused by atmospheric perturbations. The latter analysis shows that the occurrence of wing tucks is positively associated with environmental predictors of atmospheric turbulence (table 4). As a further check, we now show that the time scale of the transients associated with wing tucking is consistent with our interpretation of the manoeuvre as a response to atmospheric turbulence.

In a fixed-wing air vehicle, changes in airspeed caused, for example, by horizontal gusts lead to phugoid oscillations [27] involving coupled changes in altitude, airspeed, pitch angle and load factor. These changes occur at an almost constant aerodynamic angle of attack [27], so the lift coefficient (*C*_L_) remains approximately constant during phugoid oscillation. Although the gust response of a bird is likely to be more complicated than this, we would expect the bird's rigid body response to horizontal gusts to operate on a time scale similar to that of a phugoid oscillation. Lanchester's phugoid approximation [27] treats the motion as a conservative interchange between kinetic and potential energy, with period *T*_p_ given by4.1
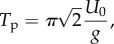
where *U*_0_ is the equilibrium airspeed, and where *g* is gravitational acceleration. With *U*_0_ ≈ 14.5 ms^−1^, as estimated from our airspeed data (see Instrumentation), the predicted phugoid period for our bird is *T*_p_ ≈ 6.5 s, which agrees quite closely with the time scales of the transients visible in figure 5. These transients appear to represent one half-period of oscillation before the wing tuck movement, and another half-period of oscillation after—each lasting approximately 3.5 s. The predicted time scale of the phugoid is therefore comparable to the time scale of the observed gust response. It is interesting to note that the duration of the wing tuck movement itself (0.35 s) is an order of magnitude shorter than the duration of the transients observed before and after, so that there may be expected to be comparatively little interaction between the events occurring on these two distinct time scales.

Although the time-series data for an individual tuck do not necessarily display all of the characteristics of a phugoid motion, the onboard data are consistent, in general terms, with an initial phugoid-like response to a headwind encountered between *t* ≈ −3.5 and *t* ≈ −0.7 s. This would explain the initial increase in airspeed that occurs in the lead up to the tuck (figure 5*c*) and the consequent increase in load factor (figure 5*a*) as well as the slow accompanying changes in pitch rate (figure 5*b*). Immediately prior to the wing tucking movement, however, the load factor starts to drop precipitously (figure 5*a*). The rate at which this drop in load factor occurs is too high to be explained by the coincident decrease in airspeed (figure 5*c*), which is manifest in the drop in *SC*_L_ that is also visible from *t* ≈ −0.7 s (figure 5*c*,*d*). This initial drop in *SC*_L_ occurs well before the bird has begun to close its wings, and must therefore be due primarily to a decrease in lift coefficient rather than in wing area. We now consider two possible explanations of this decrease in lift coefficient in the lead up to a tuck.

One potential explanation of the drop in lift coefficient at *t* ≈ −0.7 s is that the wings stall at this time, but, in this case, we would expect to see an increase in *SC*_L_ immediately beforehand, as the lift coefficient approaches its maximum at the point of stall. In fact, *SC*_L_ shows no sign of increasing prior to the tuck (figure 5*d*). Furthermore, as we now show, the numerical value of *SC*_L_ at *t* ≈ −0.7 s is too small (*SC*_L_ < 0.3) for the stall hypothesis to be credible. Making the reasonable assumption that the tail contributes either positive lift or no net lift in soaring, we know that 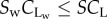, where *S*_w_ is the total area of the wings and 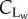 is their mean section lift coefficient. Assuming that the wings were held fully outstretched with area *S*_w_ = 0.54 m^2^ prior to tucking (table 1), we can therefore conclude that they were operating with a mean section lift coefficient of 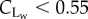. The theoretical mean section lift coefficient of an ideal elliptically loaded wing is predicted by the higher-order lifting line theory of Van Dyke [28] as4.2
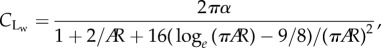
where 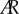 is aspect ratio, and where the angle of attack *α* is in radians (see [29] for discussion of this asymptotic approximation, which is accurate to within 1% of the exact numerical solution at 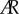 ≥ 2.55, in the context of soaring birds). Most wings stall at an angle of attack *α* ≈ 15°. Hence, given an aspect ratio of 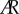 = 6.7 in soaring (table 1), we would predict a maximum attainable mean section lift coefficient of 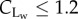. Our estimated value of 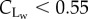 is therefore less than half that which would be expected if the wings were fully outstretched with all sections operating close to stall. This factor of two difference is much too large to be explained by any error in the assumed wing area, or by deviations from elliptic loading. Specifically, if the wings had been operating at their predicted stall limit of *C*_L_ = 1.2, then they would need to have been operating at less than half of their maximum area in order to produce the observed value of *SC*_L_, which is completely inconsistent with our ground-based video data (figure 1*a* and the electronic supplementary material, video S1). Hence, although we cannot exclude the possibility that some sections of the wing may have been stalled, our data allow us to demonstrate unequivocally that most of the wing must have been operating far from stall.

A second possible explanation of the drop in lift coefficient at *t* ≈ −0.7 s is that the angle of attack of the wings decreases at this time. Setting 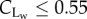 and 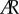 = 6.7 in equation (4.2) and solving for *α*, we estimate that the wings would have been operating at a mean angle of attack *α* ≤ 7° at *t* ≈ −0.7 s. Hence, given that the load factor halves between *t* ≈ −0.7 s and the point at which the wings begin to close (figures 5 and 7), and given that lift coefficient is proportional to angle of attack in equation (4.2), this drop in load factor could in principle have been caused by a halving of the angle of attack. Thus, a decrease in angle of attack of less than 4° would be sufficient to explain the observed drop in load factor. This comparatively small change in angle of attack might, in principle, have been caused either by the nose-down pitching motion that occurs at *t* ≈ −0.7 s (figure 5*c*), or by a hypothetical decrease in the angle of incidence of the wings relative to the body, or by an atmospheric perturbation such as a downdraft. Our data do not allow us to distinguish between these three possibilities, but we can safely conclude in the light of the above that the wings begin to tuck after experiencing a sharp loss of lift caused by a comparatively small decrease in angle of attack. Given that this loss of lift follows a transient surge in lift associated with a headwind, it is reasonable to suppose that it is ultimately the result of the same atmospheric perturbation. In summary, we conclude that wing tucks are a response to atmospheric perturbations comprising a headwind, possibly—but not necessarily—followed by a downdraft.

### 4.2. Mechanics of wing tucking

**Figure 8.**
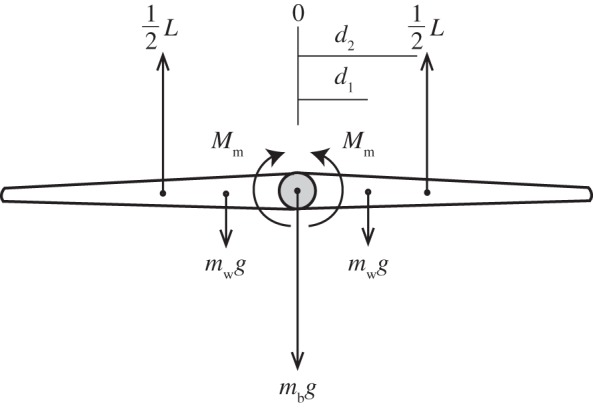
Model of the forces and moments acting on the bird's wings and body. Two rigid wings of mass *m*_w_ are assumed to be connected to the body at a single pivot point at which the mass of the body *m*_b_ is concentrated. Each wing generates half of the total lift *L*, which acts at the centre of pressure. The lengths *d*_1_ and *d*_2_ measure the distance from the wing's pivot to the wing's centre of mass and to the wing's centre of pressure, respectively. *M*_m_ is an applied musculoskeletal moment, signed positive in the direction of wing elevation.

Wing tucking represents a disturbance from an equilibrium in which the wings are held outstretched, and in order to understand the mechanics of wing tucking it is necessary to understand this equilibrium. The simplest possible model of the problem that nevertheless captures the essential physics involves treating the wings as a pair of rigid elements of mass *m*_w_, connected by a pivot at which the mass of the body *m*_b_ is concentrated (figure 8). The centre of mass of each wing is located at a distance *d*_1_ from the pivot. Each wing is assumed to produce an aerodynamic force *L*/2 equal to half of the total lift, which acts at the centre of pressure, located at a distance *d*_2_ from the pivot. Because inboard portions of a bird's wing are much heavier per unit area than outboard portions of the wing, the centre of pressure will always lie outboard of its centre of mass such that *d*_2_ > *d*_1_. For the sake of simplicity, we will consider only the equilibrium condition in which the wings are horizontal, so that the lift force can be assumed to act vertically. Considering each wing separately, the net moment (*M*) acting on the wing at equilibrium, resolved at the wing pivot, can then be expressed as4.3
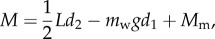
where *M*_m_ is a musculoskeletal moment, and where the moments are signed positive in the direction of wing elevation. At equilibrium, the total forces and moments acting on the wing are zero, such that *L* − *mg* = 0 and *M* = 0, where *m* = *m*_b_ + 2*m*_w_ is the total mass of the bird. Substituting these identities into equation (4.3), the musculoskeletal moment that must be generated by the bird at equilibrium is4.4
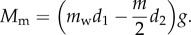
Given that *d*_2_ > *d*_1_ and *m* > 2*m*_w_, it follows that *M*_m_ < 0 at equilibrium, and hence that there must always be a musculoskeletal moment acting to pull the wing downwards in opposition to the lift produced at equilibrium (see also [30–34]).

In principle, this musculoskeletal moment could be provided either by a skeletal locking mechanism, as is known to occur in albatrosses (Diomedeidae) [33,35], or by tetanic contraction of the flight muscles which act to depress the wing. No skeletal locking mechanism is yet known to occur in raptors (Accipitriformes), but the postural muscles of many soaring birds are known to consist of slow tonic and slow twitch muscle fibres, which are specialized for sustained contraction and have a higher fatigue resistance than the fast twitch fibres found in muscles used for flapping [36]. Among the main flight muscles, the pectoralis muscle serves to depress the wings, and therefore has the appropriate action to provide the musculoskeletal moment needed to pull the wing downwards in opposition to the lift produced at gliding equilibrium [30,32,34]. Indeed, owing to its short lever arm, the pectoralis muscle has been suggested to sustain a load of at least six times body weight at gliding equilibrium [31,32,34]. It is therefore noteworthy that slow tonic fibres are present in the deep pectoralis of some birds, but only in those species which soar for extended periods [36]. This is consistent with the long-held view that the anatomical subdivision of the pectoralis in soaring birds reflects the adaptation of one of its layers for holding the wings level in gliding flight [30]. In fact, the picture is more complicated than this, because the net musculoskeletal force acting to hold the wing in place in gliding is produced by multiple musculoskeletal elements, with the ventromedial force of the pectoralis balanced laterally by the dorsomedial force of the acrocoracohumeral ligament and a lateral push from the glenoid [34].

Given that wing tucking follows a drop in aerodynamic loading (see Results), it is likely that the wings are pulled down because the musculoskeletal moment produced by the pectoralis and other flight muscles does not decrease as rapidly as the opposing aerodynamic moment. Although we cannot exclude the possibility that the bird might actively tuck its wings, in the sense of issuing a direct motor command to do so, it is perfectly plausible that wing tucks might arise passively. This is because the pectoralis is always under tension when the wings are level—even when the muscle is fully relaxed [37]. Consequently, there will always be a net musculoskeletal moment acting to pull the wings down, unless the wing elevator muscles are actively commanded to oppose this. It follows that the wings are bound to tuck under a complete loss of aerodynamic loading, unless the bird not only relaxes its wing depressor muscles, but also engages its wing elevator muscles. The same must be true of any partial loss of aerodynamic loading where the moment owing to the passive tension in the pectoralis exceeds the moment owing to the remaining aerodynamic load.

## 5. Conclusion

An obvious limitation of our study is that the data pertain to a single captive bird. Although we can draw no conclusions about the wider significance of wing tucking in this species from our data, we note that wing tucking behaviour with qualitatively indistinguishable kinematics can be observed in videos of migrating steppe eagles posted in online public video archives [38,39]. We draw attention to this footage principally to show that wing tucking is not in itself an unusual behaviour for this species, so that there is no reason to suppose that the behaviour that we have observed in our captive bird is a stereotypy or other abnormal phenomenon. Moreover, although the details of the muscle actions that are involved are unknown, the underlying physics of this kinematically similar behaviour in wild birds is unlikely to differ greatly from that which we have analysed in detail here for our captive bird. Here, we have shown specifically that the occurrence of wing tucks in our captive steppe eagle is not a deliberate mechanism for gaining airspeed prior to a flat glide or dive [10], nor an active mechanism for restoring pitch equilibrium following a gust perturbation [8]. Instead, we have provided evidence that this wing tucking behaviour is simply a response to atmospheric turbulence, just as we hypothesized previously in [17]. Specifically, our inertial data confirm that the wings only begin their tucking movement *after* experiencing a substantial loss of aerodynamic lift caused by a decrease in aerodynamic angle of attack. Logically, this *must* be because the applied musculoskeletal moment acting to pull the wing down temporarily exceeds that required to balance the aerodynamic moment acting to lift the wing up. Normal wing loading is recovered soon after the wings are reset to their equilibrium position, but it remains unclear whether the fact that the wings do tuck serves to alleviate the gust in any useful way. Nevertheless, given that soaring necessarily involves flying in turbulent conditions, the ability to deal with the adverse effects of turbulence would certainly be an important evolutionary adaptation for soaring birds. We speculate that supporting a jointed wing by using the flight muscles to resist the aerodynamic moments associated with soaring flight—with all of the energetic costs that this entails—serves to damp atmospheric turbulence, rather like the suspension of a car. It remains an open question whether wing tucking is an intrinsic part of this damping mechanism, or whether it instead represents a bifurcation in the dynamics caused when the system is pushed outside of its normal working range.

## Data accessibility

The datasets supporting this article have been uploaded as part of the electronic supplementary material.

## Supplementary Material

Dataset

## Supplementary Material

Electronic Supplementary Material
