# Supplementary material for: Wing tucks are a response to atmospheric turbulence in the soaring flight of the steppe eagle Aquila nipalensis
Source: J R Soc Interface. 2014 Dec 6;11(101):20140645. doi: 10.1098/rsif.2014.0645 (PMC4223896; doi:10.1098/rsif.2014.0645)
Supplement: Electronic Supplementary Material [file rsif20140645supp2.pdf]

# Wing tucks are a response to atmospheric turbulence in the soaring flight of the Steppe Eagle *Aquila nipalensis*

## Electronic Supplementary Material

Kate V. Reynolds, Adrian L. R. Thomas, Graham K. Taylor

Department of Zoology, University of Oxford,
Oxford, OX1 3PS, UK

## Supplementary Methods

In the absence of measurement error, the accelerations $\{a_x, a_y, a_z\}$ sensed at an arbitrary point on a rigid body are given by the equations:

$$
\begin{aligned}
a_x &= \alpha_x + (-r^2 - q^2)x + (-\dot{r} + pq)y + (\dot{q} + pr)z \\
a_y &= \alpha_y + (\dot{r} + pq)x + (-r^2 - p^2)y + (-\dot{p} + qr)z \\
a_z &= \alpha_z + (-\dot{q} + pr)x + (\dot{p} + qr)y + (-p^2 - q^2)z
\end{aligned}
$$

where $\{\alpha_x, \alpha_y, \alpha_z\}$ is the acceleration at the centre of mass, $\{p, q, r\}$ is the angular velocity, $\{\dot{p}, \dot{q}, \dot{r}\}$ is the angular acceleration, and $\{x, y, z\}$ is the displacement of the IMU from the centre of mass (Stengel, 2004). These equations are linear in $\{x, y, z\}$, and it is therefore possible to estimate the mean displacement $\{\bar{x}, \bar{y}, \bar{z}\}$ over all $i \in 1 \ldots n$ samples for a given flight as the least squares solution

to the matrix equation:

$$\begin{bmatrix} a_{x,1} \\ a_{y,1} \\ a_{z,1} \\ \vdots \\ a_{x,n} \\ a_{y,n} \\ a_{z,n} \end{bmatrix} = \begin{bmatrix} 1 & 0 & 0 & (-r_1^2 - q_1^2) & (-\dot{r}_1 + p_1 q_1) & (\dot{q}_1 + p_1 r_1) \\ 0 & 1 & 0 & (\dot{r}_1 + p_1 q_1) & (-r_1^2 - p_1^2) & (-\dot{p}_1 + q_1 r_1) \\ 0 & 0 & 1 & (-\dot{q}_1 + p_1 r_1) & (\dot{p}_1 + q_1 r_1) & (-p_1^2 - q_1^2) \\ \vdots & \vdots & \vdots & \vdots & \vdots & \vdots \\ 1 & 0 & 0 & (-r_n^2 - q_n^2) & (-\dot{r}_n + p_n q_n) & (\dot{q}_n + p_n r_n) \\ 0 & 1 & 0 & (\dot{r}_n + p_n q_n) & (-r_n^2 - p_n^2) & (-\dot{p}_n + q_n r_n) \\ 0 & 0 & 1 & (-\dot{q}_n + p_n r_n) & (\dot{p}_n + q_n r_n) & (-p_n^2 - q_n^2) \end{bmatrix} \begin{bmatrix} \bar{\alpha}_x \\ \bar{\alpha}_y \\ \bar{\alpha}_z \\ \bar{x} \\ \bar{y} \\ \bar{z} \end{bmatrix} + \begin{bmatrix} \delta_{x,1} \\ \delta_{y,1} \\ \delta_{z,1} \\ \vdots \\ \delta_{x,n} \\ \delta_{y,n} \\ \delta_{z,n} \end{bmatrix}$$

where $[\delta_{x,1}, \delta_{y,1}, \delta_{z,1}, \ldots, \delta_{z,n}]^{\mathrm{T}}$ is a vector of residuals whose sum of squares is minimized by the solution $[\bar{\alpha}_x, \bar{\alpha}_y, \bar{\alpha}_z, \bar{x}, \bar{y}, \bar{z}]^{\mathrm{T}}$, with T as the matrix transpose.

We solved this matrix equation separately for each flight, using the instantaneous acceleration and instantaneous angular velocity sensed by the IMU as $\{a_{x,i}, a_{y,i}, a_{z,i}\}$ and $\{p_i, q_i, r_i\}$, respectively, and using central differencing to estimate the instantaneous angular acceleration $\{\dot{p}_i, \dot{q}_i, \dot{r}_i\}$ from the instantaneous angular velocity. The fitted parameters $\{\bar{\alpha}_x, \bar{\alpha}_y, \bar{\alpha}_z\}$ estimate the mean acceleration at the centre of mass over the whole flight, and can be interpreted as regression intercepts. The fitted parameters $\{\bar{x}, \bar{y}, \bar{z}\}$ estimate the mean displacement of the IMU from the centre of mass, and can be interpreted as regression slopes. In effect, then, we are regressing the acceleration sensed by the IMU on those combinations of angular velocity and angular acceleration that are expected to be linearly related to the sensed acceleration by virtue of the IMU's unknown—but still possible to estimate—displacement from the centre of mass.

It can be seen by comparing this matrix equation with the preceding set of simultaneous equations that the instantaneous acceleration at the centre of

mass $\{\alpha_{x,i}, \alpha_{y,i}, \alpha_{z,i}\}$ is estimated as:

$$\begin{bmatrix} \alpha_{x,i} \\ \alpha_{y,i} \\ \alpha_{z,i} \end{bmatrix} = \begin{bmatrix} a_{x,i} \\ a_{y,i} \\ a_{z,i} \end{bmatrix} - \begin{bmatrix} (-r_i^2 - q_i^2) & (-\dot{r}_i + p_i q_i) & (\dot{q}_i + p_i r_i) \\ (\dot{r}_i + p_i q_i) & (-r_i^2 - p_i^2) & (-\dot{p}_i + q_i r_i) \\ (-\dot{q}_i + p_i r_i) & (\dot{p}_i + q_i r_i) & (-p_i^2 - q_i^2) \end{bmatrix} \begin{bmatrix} \bar{x} \\ \bar{y} \\ \bar{z} \end{bmatrix}$$

This will tend to underestimate the required correction, because the estimates of the mean displacement of the IMU from the origin of the bird's axis system are effectively regression slopes, so will be asymptotically biased towards zero in the presence of any error in the measurements of angular velocity and angular acceleration that were used to form the predictor variables. In fact, the IMU was estimated to be $\leq 29$ mm from the centre of mass in the $x$- and $y$-directions, and $\leq 37$ mm above the centre of mass in the $z$-direction. These estimates seems reasonable, if a little on the low side perhaps. Nevertheless, the effect of making this correction will be to reduce a known source of systematic error in our measurements of the bird's acceleration, and for this reason alone, the correction is worthwhile. The same correction should also serve to reduce measurement errors associated with rotation of the IMU with respect to the bird's axis system, as occur, for example, during flapping (i.e. to correct for accelerations sensed by the IMU as a result of the bird's non-rigid body motion).

# References

Stengel, R. F. 2004 *Flight dynamics,* Princeton: Princeton University Press.
